# Human-AI teaming: leveraging transactive memory and speaking up for enhanced team effectiveness

**DOI:** 10.3389/fpsyg.2023.1208019

**Published:** 2023-08-04

**Authors:** Nadine Bienefeld, Michaela Kolbe, Giovanni Camen, Dominic Huser, Philipp Karl Buehler

**Affiliations:** ^1^Work and Organizational Psychology, Department of Management, Technology, and Economics, ETH Zürich, Zurich, Switzerland; ^2^Institute of Intensive Care Medicine, University Hospital Zurich, Zurich, Switzerland; ^3^Department of Intensive Care Medicine, Cantonal Hospital Winterthur, Winterthur, Switzerland

**Keywords:** human-AI teams, transactive memory systems, speaking up, explainable artificial intelligence / XAI, healthcare teams, behavioral observation, interaction analysis, team performance

## Abstract

In this prospective observational study, we investigate the role of transactive memory and speaking up in human-AI teams comprising 180 intensive care (ICU) physicians and nurses working with AI in a simulated clinical environment. Our findings indicate that interactions with AI agents differ significantly from human interactions, as accessing information from AI agents is positively linked to a team’s ability to generate novel hypotheses and demonstrate speaking-up behavior, but only in higher-performing teams. Conversely, accessing information from human team members is negatively associated with these aspects, regardless of team performance. This study is a valuable contribution to the expanding field of research on human-AI teams and team science in general, as it emphasizes the necessity of incorporating AI agents as knowledge sources in a team’s transactive memory system, as well as highlighting their role as catalysts for speaking up. Practical implications include suggestions for the design of future AI systems and human-AI team training in healthcare and beyond.

## 1. Introduction

The rapid technological advances of recent years and months bring forth increasingly powerful AI agents that are able to assist clinicians in the assessment of critically ill patients and largely reduce the burden on medical staff (Moor et al., 2023). Current evaluations of human-AI collaboration focus predominantly on human-factors-related issues and dyadic interactions between one human and one AI agent (Lai et al., 2021; Knop et al., 2022), thus neglecting the fact that most healthcare work is conducted in larger inter-disciplinary teams (Dinh et al., 2020).

Interactions in human-AI teams, where multiple humans and AI agents interact dynamically and interdependently are bound to be more complex than dyadic ones, yet to date, such interactions have not been sufficiently investigated. This is especially true for real teams collaborating with actual AI agents as past research has mainly used “make-believe” AI agents (i.e., humans pretending to be an AI) in laboratory settings (McNeese et al., 2021; Endsley et al., 2022; O’Neill et al., 2022).

In healthcare, ineffective human-AI teaming could have life-or-death consequences. Consider, for instance, a team’s failure to access or misinterpret information from an AI agent that is crucial for diagnosing a critically ill patient. The black-box nature of today’s AI agents—which lack explainability because they discern patterns in data without pre-set rules— makes collaboration with AI agents particularly challenging (Lecun et al., 2015; Wiens et al., 2019). To enable effective human-AI team collaboration in healthcare, it is crucial to imbue AI agents with optimal levels of explainability, interpretability, and plausibility, at least regarding the nature of knowledge employed—such as its source, patient cohort, and clinical context (Kundu, 2021; Bienefeld et al., 2023).

A team’s transactive memory system (TMS) (Lewis and Herndon, 2011) could help team members remember and retrieve distributed knowledge in the team, including the knowledge held by AI. Building TMS in human-AI teams may be difficult due to the black-box problem outlined above, making it practically impossible to “know what the AI knows” (Durán and Jongsma, 2021). Also, since AI agents cannot (yet) proactively communicate their “view of the world,” unless a human team member speaks up on their behalf, communication breakdowns and performance losses are inevitable (Yan et al., 2021).

To help reduce these risks and to close the gap in knowledge about human-AI team interaction in healthcare, we investigate TMS and speaking up behavior in *N* = 180 intensive care unit (ICU) physicians and nurses collaborating with an AI agent in a simulated, yet realistic clinical setting. We draw on the team science literature (see e.g., Kozlowski and Ilgen, 2006 for an overview) to attain this goal and define *human-AI teams* as (a) two or more human team members interacting with one or more AI agents; (b) having interdependencies regarding workflow, goals, and outcomes, and (c) contributing to shared team goals.

### 1.1. Transactive memory systems in healthcare teams

**Figure 1:**
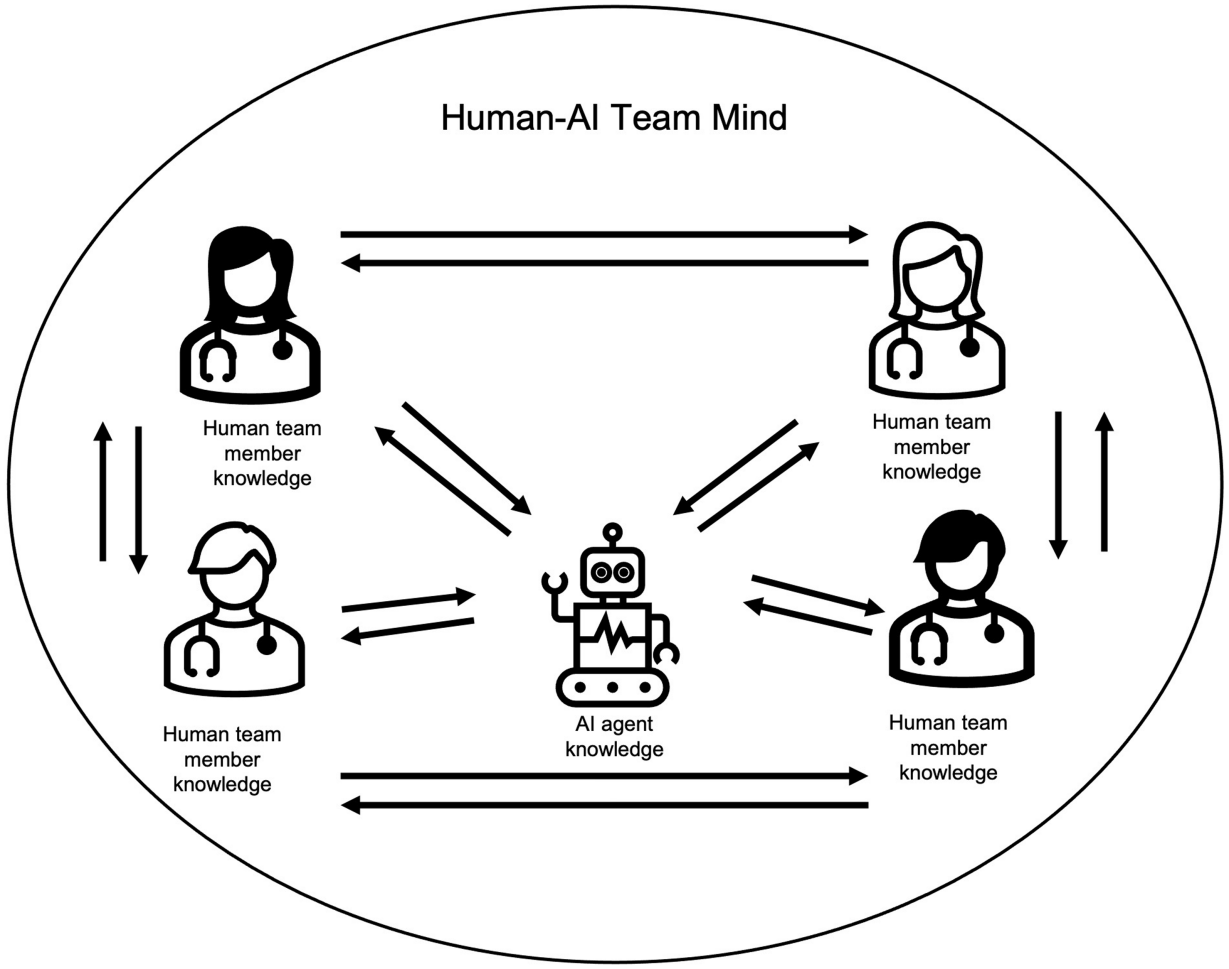
Visualization of TMS and speaking up interactions in human-AI teams.

Transactive memory systems (TMS) are defined as the “group-level knowledge sharing and memory system for encoding, storing, and retrieving information from different knowledge areas in a group” (Yan et al., 2021, p. 52). As shown in Figure 1, “knowing what other team members know” and accessing this knowledge when needed, helps assemble the different pieces of distributed group knowledge into one coherent “group mind.” This group mind is associated with team effectiveness (DeChurch and Mesmer-Magnus, 2010). Since AI agents may hold mission-critical information, their knowledge should be included in a team’s TMS, which has, however, not yet been researched in human-AI teams.

Tapping into and sharing distributed group knowledge is key for adequate hypothesis-building and decision-making in teams (Palazzolo, 2017), but can be challenging, particularly in diverse and/or hierarchical teams (Ren and Argote, 2011). Furthermore, group members tend to exchange more “shared” (i.e., known by all members) than “unique” (i.e., known only to individual group members) knowledge, which gets further strengthened via confirmation by others in some kind of a vicious circle (Stasser and Titus, 1985; Lewis and Herndon, 2011; Boos et al., 2013). This is problematic and can negatively impact performance because good decisions, e.g., finding the correct diagnosis, depend on a team’s ability to choose the most viable option amongst a diverse range of hypotheses (Mesmer-Magnus and DeChurch, 2009; Kämmer et al., 2017). Accessing knowledge from AI agents might provide a way out of this vicious circle because AI agents are not affected by social group dynamics and—based on their immense data storage and analytical capabilities (Moor et al., 2023)—are likely to hold unique knowledge other team members do not possess. Based on these considerations, we propose the following hypotheses:

*Hypothesis 1a*: In higher-performing teams, “accessing knowledge from the *AI agent*” is more likely followed by “developing new hypotheses” than in lower-performing teams.

*Hypothesis 1b*: In higher-performing teams, “accessing knowledge from a *human team member*” is more likely followed by “developing new hypotheses” than in lower-performing teams.

### 1.2. Speaking up in healthcare teams

Speaking up (or voice) is defined as “informal and discretionary communication by an employee of ideas, suggestions, concerns, information about problems […] to persons who might be able to take appropriate action […]” (Morrison, 2014, p. 174). Numerous positive effects such as enhanced decision-making, improved learning, and higher team performance are associated with people’s willingness to speak up (Edmondson, 2003; Pfrombeck et al., 2022; Weiss and Zacher, 2022; Morrison, 2023). However, speaking up and respective listening remains challenging because people fear (1) personal embarrassment and doubts about how valid their knowledge is, (2) social repercussions such as creating conflict with other team members or not being a good team player, and because consequently, they suffer from (3) social dynamics impeding positive speaking up experiences (Noort et al., 2019; Long et al., 2020; Sessions et al., 2020).

Because the hurdles to speaking up are predominantly social, team members may find it easier to speak up based on information coming from an AI agent rather than from a human colleague. If people speak up “on behalf of the AI,” they may not be as afraid to be personally blamed or lose face. Since speaking up behavior, in general, helps correct faulty decisions or a wrong course of action, in Hypotheses 2 a and b, we assume that speaking up based on knowledge received from the AI and/or other human team members will be associated with higher team performance.

*Hypothesis 2a*: In higher-performing teams, “accessing knowledge from the *AI agent*” is more likely followed by “speaking up” than in lower-performing teams.

*Hypothesis 2b*: In higher-performing teams, “accessing knowledge from a *human team member*” is more likely followed by “speaking up” than in lower-performing teams.

## 2. Methods

### 2.1. Participants

Resident and attending physicians and nurses from the Institute of Intensive Care Medicine at a large teaching hospital in Switzerland were invited to participate in this study as they took part in their yearly team-based simulation training. Training took place during work hours and participants received education credits (no other remuneration). Study participation was voluntary and independent of the training. Full anonymity was granted and written consents were given by participants with the possibility to opt out at any time and without any repercussions. *N* = 180 participants chose to participate in the study and were randomly assigned to 45 interdisciplinary 4-person teams. Each physician or nurse acted according to their actual function and, although some participants were acquainted, nobody had previously worked together in the same team.

### 2.2. Study design and procedure

In this prospective observational study, 180 ICU physicians and nurses collaborated with an AI agent to diagnose and provide medical treatment to a simulated patient suffering from a life-threatening condition. The simulated setting was chosen to create a realistic yet controlled environment without putting real patients at risk (Cheng et al., 2016). For this purpose, a fully equipped, state-of-the-art simulation facility including an advanced simulation training mannequin with interactive patient features (vital signs, pulse, heartbeat, chest movements) was used (SimMan3G^®^, Laerdal, Stavanger, Norway). Four simulation training medical faculty members (one attending physician and three nurses, all specialized in intensive care medicine) led the simulation training and were blinded to the hypotheses. They provided an introduction to the simulated setting, learning objectives, and procedures to establish a psychologically safe learning environment (Rudolph et al., 2014). Each scenario was audio and video recorded to enable video-based debriefing—a standard practice at the simulation center (Zhang et al., 2019). Participants were familiar with this practice due to prior participation in simulation training, thus minimizing the Hawthorne effect (Wickström and Bendix, 2000; Soukup et al., 2021). To minimize observer bias, significant time (>8 h) and effort was invested into behavioral coding training and specifying each code with specific examples. One major in psychology and health sciences—blinded to the hypotheses—coded the entire data set. To determine interrater reliability, 10% of the data were randomly chosen and coded by a psychology minor, also blinded to the hypotheses and also having undergone behavioral observation training. As displayed in Table 1, Cohen’s kappa values represent substantial strength of agreement (Landis and Koch, 1977).

**Table 1:** Behavior codes, descriptive statistics, and independent *t*-tests for study variables for lower- and higher-performing teams.

				Lower performing teams		Higher performing teams				95% CI	
Behavior	Definition	Examples	*κ* (ICC) ^d^	*M*	*SD*	*M*	*SD*	*t* ^e^	*p*	*LL*	*UL*
Accessing knowledge from a *human team member*	^a^Searching for information from a human team member when knowing who has it.	Did [the patient] have bradycardia already when you got here?	0.87	17.93	5.48	22.30	9.58	−1.63	0.110	−1.14	0.11
Accessing knowledge from the *AI agent*	^b^Searching for information when knowing that the AI agent has it.	Non-verbal behavior. Searching for specific information stored in the AI agent by opening and closing tabs on the computer screen, analyzing data, and looking for patterns in the data, often combined with adjusting certain ventilation parameters.	0.91	19.13	10.60	16.47	9.69	0.84	0.404	−0.35	0.88
Developing new hypotheses	^a^Articulating ideas about what could be the correct diagnosis based on information received or summarizing all the available information.	Hmm, SpO2 and PetCo2 are getting really low […] Maybe it could be air trapping since [the patient] has COPD [Chronic obstructive pulmonary disorder]?	0.83	11.60	8.59	14.03	8.26	−0.91	0.363	−0.91	0.33
Interacting with non-AI technologies	^b^Reading indicators on a monitor screen (e.g., heart frequency) or gathering information from additional non-AI technologies (e.g., ultrasound or CPR device).	Non-verbal behavior, mostly short glances at a computer screen.	0.69	17.20	7.55	14.83	4.49	1.31	0.194	−0.21	1.04
Speaking up (doubt-focused voice)	^c^Voicing doubts or contradicting what is being said or done by other team members.	I do not think it’s that [pericardial tamponade], look, the tidals [wave-form length of breathing patterns as indicated by the AI agent] are far too low and I cannot get a clear sound on the right lung [auscultating the lungs].	0.79	4.67	2.74	5.70	3.83	−0.92	0.358	0.91	0.33
Team performance	Accuracy and timeliness of diagnosis, suitability, and quality of the medical treatment provided to the patient based on established standards in intensive care medicine and hospital best practices/guidelines.	Correct and timely diagnosis of, e.g., a pressure pneumothorax. Adequate and timely treatment, e.g., of a pressure pneumothorax (i.e., needle decompression by inserting a 14- or 16-gauge needle/Venflon into the 2nd intercostal space in the midclavicular line) and insertion of thorax drainage. Patient stabilizes after procedure (systolic blood pressure 80–140 mm Hg or MAP >50; heart rate 60–100 per minute, oxygen saturation SaO2 > 95%).	[0.87]	8.20	3.36	13.43	4.04	−4.31	0.000	−2.04	−0.67

*N* = 180 (45 teams). ^a^Definitions based on TRAWIS (Brauner, 2006, 2018); ^b^Self-developed; ^c^Definitions based on the Co-ACT coding framework (Kolbe et al., 2013). ^d^Cohen’s kappa and [ICC] values representing acceptable to good interrater agreement (Landis and Koch, 1977); ^e^Independent sample *t*-tests (two-sided) with Cohen’s d Lower (LL) and Upper (UL) 95% Confidence Interval (CI).

### 2.3. Scenarios

Each team participated in one of three standardized scenarios designed by the last author (BPK, an experienced chief physician). Scenarios were based on documented cases of real-life events in intensive care medicine. The team’s goal in each scenario consisted of diagnosing and providing treatment to a critically ill simulated patient presenting with a set of symptoms (myocardial infarction with atrioventricular block; polytrauma with pneumothorax; septic shock). At the start of the scenario, participants received realistic patient information documents drawn from real cases and detailing information on the patient’s history, medication, and symptoms. Audio and video data of the study scenarios and clinical performance measures (e.g., heart rate, pulse, blood pressure, and ventilation parameters such as SaO2, and Spo2) were recorded in real-time. After each scenario, participants took part in a video-based debriefing led by simulation faculty following the Debriefing with Good Judgment approach (Rudolph et al., 2007).

### 2.4. AI agent

AI agents are different from other technologies insofar as they can learn from vast amounts of data and possess the agency to perform tasks that were previously performed by human team members (Kaplan and Haenlein, 2019). In this study, *Autovent*^1^, a state-of-the-art auto-adaptive ventilator using complex algorithms to control patients’ ventilation cycles of inspiration and expiration was used as the AI agent. The AI agent autonomously completed the task of ventilation and weaning—a task previously performed by physicians and nurses—by “continuously extracting data from patient-specific data streams (e.g., PetCO2, SpO2, lung mechanics, and muscle activity) and personalized waveform shapes of either oxygen flow or pressure” (Autovent training manual, 2023: p. 12). To assure sufficient familiarity with the AI agent, participants needed to have worked with *Autovent* for at least 6 months to be able to participate in the study.

### 2.5. Variables

#### 2.5.1. Transactive memory in human-AI teams

TMS in human-AI teams was assessed with TRAWIS—a behavior observation instrument measuring processes that lead to the development of transactive memory by Brauner (2006, 2018). As described above, a major in psychology and health sciences with specialist training in behavioral observations and blinded to the hypotheses applied an event-sampling procedure by assigning one of four codes to the complete data set: (1) “accessing knowledge from a *human teammate*”; (2) “accessing knowledge from the *AI agent*” (self-developed); (3) “developing new hypotheses,” and (4) “monitoring/interacting with non-AI technologies” (self-developed, to distinguish interactions with the AI from other, non-AI-based technologies used in the ICU). Every distinct behavior or utterance, i.e., sense unit (Bales, 1950) was coded in the following sequence: (A) actor; (B) code; (C) receiver, and (D) timing (beginning, end, and duration in seconds). Interact software (Mangold, 2022) was used for behavioral coding and data analysis. Please refer to Table 1 for a detailed description of all TMS codes, examples, and Cohen’s Kappa values indicating considerable interrater agreement (Landis and Koch, 1977).

#### 2.5.2. Speaking up in human-AI teams

Speaking up behavior was assessed in the identical ways as described above using the Co-ACT framework (Kolbe et al., 2013). This framework captures a broad range of verbal and non-verbal communication and coordination behavior in acute care teams, including the variable of interest—speaking up behavior (Kolbe et al., 2012; Weiss et al., 2017; Lemke et al., 2021). Because we were interested especially in doubt-focused voice (Weiss et al., 2014), speaking up was coded whenever a team member spoke up with information or knowledge that contradicted what was being said or done after accessing knowledge from either the AI agent or another human team member. Please refer to Table 1 for a detailed description of the speaking up code with an example and Cohen’s Kappa values indicating considerable interrater agreement (Landis and Koch, 1977).

#### 2.5.3. Clinical performance assessment

In a Delphi-like consensus-building process (Hasson et al., 2000), three authors (BPK, HD, CG) all specialized in intensive care medicine with more than 10 years of clinical experience developed a case-specific clinical performance measure including 29–34 items per scenario. These items are related to the specific medical condition, the accuracy and timeliness of diagnosis, and the effectiveness of selected treatment options based on established standards in intensive care medicine and best medical practice according to the Competency-Based Training program in Intensive Care Medicine for Europe and other world regions (CoBaTrICE describing 102 competencies divided into 12 domains European Society for Intensive Care Medicine, 2023). Two attending physicians (HD & CG) blinded to the hypotheses yet familiar with the hospital’s best practices and standard operating procedures then independently coded the complete set of audio and video data (*N* = 180 ICU physicians and nurses split into 45 teams). They applied the checklist-based team performance measure to code each video file while also considering patient data from vital signs with target values (e.g., systolic blood pressure 80–140 mm Hg or MAP >50; heart rate 60–100 per minute, oxygen saturation SaO2 > 95%). Interrater reliability was calculated on the complete data set using the intraclass correlation coefficient, which resulted in a satisfactory reliability measure (Landis and Koch, 1977) (see Table 1).

#### 2.5.4. Control variables

Demographic information included age (in years), sex (male–female), professional role (nurse, resident physician, attending physician), work experience since graduation from medical/nursing school (in years), and experience working with the AI agent (in months).

### 2.6. Data analysis

Due to the variation in the length of the simulated scenarios, we divided the number of codes per category by the length of the video in minutes and then multiplied by 20 for standardization. To compare higher- versus lower-performing teams in terms of how frequently (i.e., number of occurrences) they exhibited the coded behaviors, we conducted a series of independent *t*-tests (two-sided) for each of the five behaviors. For this purpose, we previously split the data by the median, creating two groups (higher- vs. lower-performing teams) (Stout et al., 1999; Waller et al., 2004). To test the hypotheses, a lag sequential analysis was conducted (Bakeman and Gottman, 1997; Bakeman and Quera, 2011) for both higher- and lower-performing teams. This method involves generating *z*-values from frequencies of each interaction sequence to determine which temporal patterns occur more or less frequently than expected. Any *z*-values larger than 1.96 or smaller than −1.96 indicate a statistically significant interaction pattern. Positive *z*-values indicate a facilitating effect of behavior A on a subsequent behavior B, and negative *z*-values indicate an inhibitory effect of behavior A on subsequent behavior B. In this study, only behavior B directly following behavior A (lag 1) was of interest. To calculate the required event sequences based on the total number of coded events (*N* = 9,850) for 5 codes, the formula developed by Bakeman and Gottman (1997) was used. Interact software (Mangold, 2022) was then used to compute two interaction matrices with *z* values for teams above/below the performance measure median.

## 3. Results

In total, *N* = 180 ICU nurses and physicians participated in this study (45 teams). 101 participants were female (56.1%), 79 were male (43.9%) and the average age was 38.10 (*SD* = 7.53). The average experience working as a physician or nurse was 11.85 years (*SD* = 8.10) and the average experience working with the AI agent was 2.89 years (*SD* = 1.90).

Out of the 45 teams, 22 teams (48.89%) were above the median (i.e., higher-performing), and 23 teams (51.11%) were below the median (lower-performing). As shown in Table 1, the results of the independent t-tests (two-sided) for each of the five behaviors revealed no significant differences between higher- and lower-performing teams in terms of how frequently they exhibited each of the five behaviors.

To test our hypotheses, we conducted lag sequential analyses to examine the behavioral reactions to “accessing knowledge from *the AI agent*” versus “accessing knowledge from *human team members*.”

**Figure 2:**
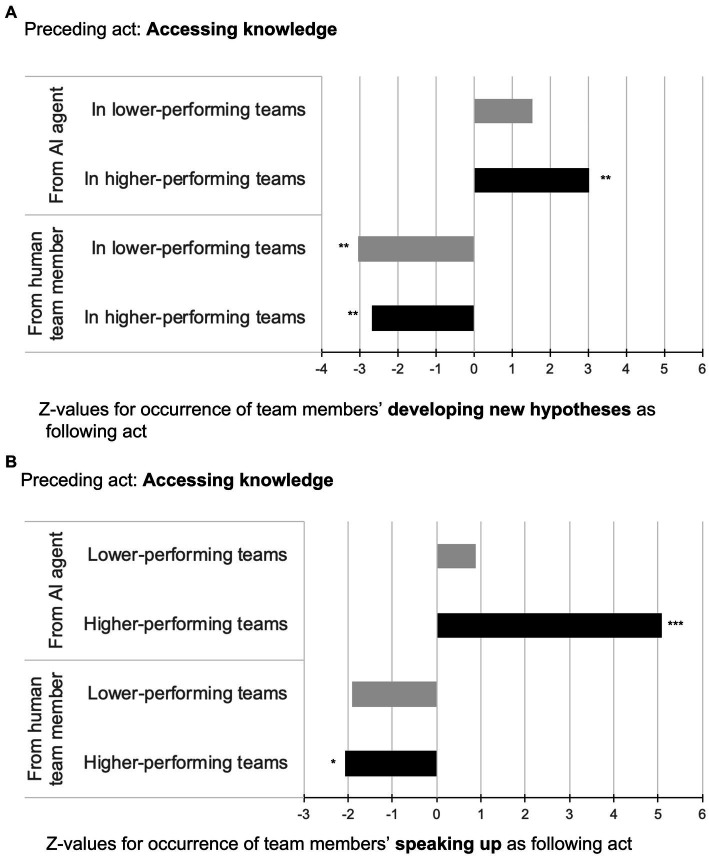
Illustration of sequential analyses for accessing knowledge from AI agents vs. from human team members followed by developing new hypotheses **(A)** and speaking up **(B)** in higher- and lower-performing teams.

Hypothesis 1a stated that in higher-performing teams, “accessing knowledge from the *AI agent*” was more likely followed by “developing new hypotheses” than in lower-performing teams. As depicted in Figure 2A (upper part), this hypothesis was supported by comparing the interaction sequences of “accessing knowledge from the *AI agent*” on “developing new hypotheses” for higher-performing teams (*z* = 3.01, *p* = 0.004) versus lower-performing teams (*z* = 1.55, *p* = 0.012).

Hypothesis 1b stated that in higher-performing teams, “accessing knowledge from a *human team member*” was more likely followed by “developing new hypotheses” than in lower-performing teams. As shown in Figure 2A (lower part), this hypothesis was not supported since “accessing knowledge from a human team member”—though significant—was *negatively* associated with the target behavior of “developing new hypotheses.” This result was observed in both higher- and lower-performing teams thus indicating a suppressing effect from the behavior “accessing knowledge from a *human team member*” on “developing new hypotheses” independent of team performance (−2.68, *p* = 0.007 for higher-performing teams; *z* = −3.03, *p* = 0.004 for lower-performing teams).

Hypothesis 2a stated that in higher-performing teams, “accessing knowledge from the *AI agent*” was more likely followed by “speaking up” than in lower-performing teams. As depicted in Figure 2B (upper part), this hypothesis was supported by comparing the interaction sequences of “accessing knowledge from the *AI agent*” on “speaking up” for higher-performing teams (*z* = 5.09, *p* = 0.000) versus lower-performing teams (*z* = 0.87, *p* = 0.273).

Hypothesis 2b stated that in higher-performing teams, “accessing knowledge from a *human team member*” was more likely followed by “speaking up” than in lower-performing teams. As shown in Figure 2B (lower part), this hypothesis was not supported since “accessing knowledge from a *human team member*”—though significant—was *negatively* associated with the target behavior “speaking up.” Again, this result was observed in both higher- and lower-performing teams indicating a suppressing effect from the behavior “accessing knowledge from a *human team member*” on “speaking up” regardless of team performance (*z* = −2.06, *p* = 0.048 for higher-performing teams; *z* = −1.92, *p* = 0.063 for lower-performing teams).

## 4. Discussion

The goal of this study was to increase our understanding of how humans collaborate with AI in a team setting and how different interaction patterns relate to team effectiveness. Drawing on the team science literature, we investigated human-AI team interaction behavior relating to TMS and speaking up by observing *N* = 180 intensive care physicians and nurses as they worked with an AI agent in a simulated, yet realistic clinical environment. The results demonstrate that in higher-performing teams accessing knowledge from an AI agent is positively associated with a team’s ability to develop new hypotheses and speaking up with doubts or concerns. In contrast, accessing knowledge from a human team member appeared to be negatively associated with hypothesis-building and speaking up, regardless of team performance.

### 4.1. Theoretical contributions

Our findings contribute to research on TMS and speaking up and to team science more broadly in three ways. First, the identified interaction patterns between accessing knowledge from the AI agent versus from another human team member were notably different. This finding indicates that we cannot *per se* generalize theory on human-human team interactions to human-AI team interactions. This conclusion paves the way for abundant future research opportunities investigating the various team Input-Mediator-Output–Input (IMOI) factors summarized in the well-established IMOI model (Ilgen et al., 2005). For example, shared mental models (SMM)—i.e., “cognitive representations of reality that team members use to describe, explain, and predict events” (Burke et al., 2006, p. 1199)—could help increase our understanding of how members of human-AI teams can be aligned “on the same page.” Investigating the role of SMM in human-AI teams is an essential next step because research on human-only teams has shown that shared and accurate representations of what is going on during a team’s mission facilitates team coordination and predicts team effectiveness (DeChurch and Mesmer-Magnus, 2010).

Second, even though in this study, the task of correctly diagnosing and providing treatment to a critically ill patient could be achieved also without the knowledge of the AI agent, accessing knowledge from the AI rather than a human team member was associated with developing new hypotheses and higher team performance. Because AI agents are able to compute vast amounts of data and make predictions beyond human capabilities (Kaplan and Haenlein, 2019), they likely hold unique knowledge relevant to hypothesis building. Actively integrating AI agents as sources of knowledge within a team’s TMS could thus indicate a competitive advantage. A team’s ability to fully leverage this advantage depends on two conditions: First, team members must be able to understand how the AI’s knowledge is created. This calls for research on explainable AI (XAI) in human-AI teams, which is thus far lacking (see Bienefeld et al., 2023 for an exception). The results of this study serve as a promising foundation for future research on XAI in teams as the concept of TMS can be used to assess people’s interpretations of AI on the team level. Also, team members must calibrate their level of trust in the AI agent, i.e., finding the right balance between trusting AI too much or too little, with the former posing more serious safety concerns due to the risk of overreliance (Parasuraman and Riley, 1997). Research on trust in AI has thus far focused mainly on the human-AI dyad (Glikson and Woolley, 2020). Extending this research to the human-AI team level is thus indicated and should not only focus on how trust is established between humans and the AI agent but also consider how the presence of an AI agent may affect the trust between two or more human members of the team (e.g., a senior physician may have higher or lower trust in a junior physician depending on whether he or she collaborates with an AI agent or not).

Third, our results show that accessing knowledge from the AI agent was positively associated with speaking up, whereas the reverse pattern was found when knowledge was accessed from human team members. This suggests that people might feel more comfortable voicing concerns or expressing doubts based on information that comes from an AI agent rather than from a human team member. Future research should explore the mechanisms explaining this inclination because a better understanding of this phenomenon may provide new ways of promoting speaking-up behavior in teams more generally. The possibility of using AI to foster speaking up in teams, however, comes with one important caveat: If people were to “hide behind the technology” to speak up, their personal, equally valid doubts or concerns might get lost, or they might give up trying to overcome their social fears to enable candid communication. Researchers and healthcare practitioners should continue investing in efforts promoting speaking up both on the technological as well as on the human side, e.g., via team training and building a psychologically safe team environment (Kolbe et al., 2020; Jones et al., 2021).

### 4.2. Practical implications

The findings of this study offer multiple suggestions for the design of future AI agents. Considering the role of an AI agent as some kind of “teammate” rather than a tool, future AI agents should be designed with more advanced teaming capabilities. Human-AI teaming capabilities are defined as “the knowledge, skills, and strategies with respect to managing interdependence [between humans and AI …] such as being capable of observing one another’s state, sharing information, or requesting assistance” (Johnson and Vera, 2019, p. 18). Take for example interactions with ChatGPT (OpenAI, 2023). Only if the capabilities of the chatbot in terms of remembering previous inputs and self-correcting its own mistakes are combined with the skill of human users entering suitable prompts, can the most reliable outcomes be produced (Lee et al., 2023). As suggested by Tartaglione et al. (2021), such advanced teaming capabilities would require the AI agent to dynamically update information based on “what human team members know” including their roles and task responsibilities, which is a challenging goal. Also, equipping AI with better teaming capabilities requires AI systems that can learn “*in situ*,” i.e., systems that are able to continuously learn from new data rather than “freezing” trained algorithms once they are employed into clinical practice (as is current practice for AI agents certified as medical devices van Hartskamp et al., 2019). Nevertheless, as AI agents advance rapidly in terms of their sensing and data processing capabilities, we are hopeful that they will one day be able to proactively support human team members also in dynamic real-life settings (e.g., by prompting them to speak up with safety-critical information at the right time). Given these rapid technological developments and the fact that more and more healthcare professionals are or will be working in human-AI teams, the results of this study should also be used to train people on how to effectively interact with AI agents. The knowledge gained from this study such as how interaction patterns in human-AI teams differ from those in human-only teams in terms of TMS and speaking-up behaviors—in combination with other human-AI interaction skills—can provide healthcare professionals with a real competitive advantage.

### 4.3. Strengths and limitations

As with any study, there are various limitations to consider when interpreting the results. Observing how real human-AI teams interact “in the wild” (Klonek et al., 2019; Kolbe and Boos, 2019) is certainly a strength of this study; especially because prior research has relied on make-believe AI agents in laboratory settings (O’Neill et al., 2022). Another advantage of this study consists of our focus on micro-level lag sequential analyses, which allowed us to reveal differences in interaction patterns between human-AI agent versus human-human interactions and between higher- versus lower-performing teams. These design choices, however, limit our ability to infer the causality of effects, for which randomized controlled trial studies would be the gold standard. Also, due to patient safety concerns, we were restricted to a simulated setting. This may have introduced simulation artifacts like the Hawthorn effect (Wickström and Bendix, 2000). Although we minimized these effects by (1) selecting participants who were accustomed to being observed due to prior training experiences (2) using non-obtrusive cameras to make audio and video recordings (Soukup et al., 2021), and (3) investing significant time and effort into high-quality observer training (Kolbe and Boos, 2019), we cannot fully eliminate the potential for such biases.

Finally, our study design did not allow us to test for potential moderators such as team context, team size, task complexity, or team member personality. Given the unique, high-risk, and high-time–pressure context of a hospital ICU, one might find different team interaction patterns in low-risk, low-time–pressure situations. Other types of teams, even within healthcare, may face completely different challenges regarding their mission, thus requiring different interaction behaviors. We would also expect different ways of team interaction depending on the type and level of autonomy of the AI agent. The selection of the AI agent as one focused on ventilatory auto-adaptation may have somewhat limited team interaction possibilities. More sophisticated and generative AI agents such as future versions of large language models fine-tuned for healthcare (Cascella et al., 2023; Lee et al., 2023; Moor et al., 2023) would certainly offer new and different knowledge creation possibilities. We hope that this study may inspire future researchers to tackle these questions and to further advance the promising new field of human-AI team research in healthcare and beyond.

## Data availability statement

The datasets presented in this article are not readily available because video data cannot be made de-identifiable and therefore cannot be shared. Requests to access the datasets should be directed to n.bienefeld@gmail.com.

## Ethics statement

The studies involving human participants were reviewed and approved by ETH Zürich Ethics Committee No. EK 2019-N-190. The patients/participants provided their written informed consent to participate in this study. Written informed consent was obtained from the individual(s) for the publication of any potentially identifiable images or data included in this article.

## Author contributions

NB and PKB contributed to the study design, data analysis plan, review, and analysis of the results. NB was the principal investigator, conducted the data collection, data analysis, and implemented the study protocol. PKB provided his medical expertise to design the study scenarios, provided access to the simulation medical faculty, and participated in the development of the performance measure. MK provided access to the simulation center and technical staff. DH and GC contributed equally to assessing team performance. NB, PKB, and MK contributed to the preparation of the manuscript. All authors read and approved the manuscript.

## Funding

This work was supported by funding from the Swiss National Science Foundation NRP77 Digital Transformation Programme (Grant no. 187331). This information or content and conclusions are those of the authors and should neither be construed as the official position or policy of nor should any endorsements be inferred by the Swiss National Science Foundation. Open access funding by ETH Zurich.

## Conflict of interest

The authors declare that the research was conducted in the absence of any commercial or financial relationships that could be construed as a potential conflict of interest.

## Publisher’s note

All claims expressed in this article are solely those of the authors and do not necessarily represent those of their affiliated organizations, or those of the publisher, the editors and the reviewers. Any product that may be evaluated in this article, or claim that may be made by its manufacturer, is not guaranteed or endorsed by the publisher.

## Footnotes

^1^An acronym used to protect anonymity and non-disclosure agreements.
